# Washing obsessive-compulsive symptoms as adaptation—insights from a 3-year longitudinal cohort during the COVID-19 pandemic

**DOI:** 10.3389/fpsyg.2025.1542724

**Published:** 2025-06-17

**Authors:** Ana Daniela Costa, Catarina Raposo-Lima, Beatriz Couto, Afonso Fernandes, Mafalda Machado-Sousa, Pedro Silva Moreira, Sónia Ferreira, Maria Picó-Pérez, Pedro Morgado

**Affiliations:** ^1^Life and Health Sciences Research Institute (ICVS), School of Medicine, University of Minho, Braga, Portugal; ^2^ICVS/3B’s, PT Government Associate Laboratory, Guimarães, Braga, Portugal; ^3^Association P5 Digital Medical Centre (ACMP5), Braga, Portugal; ^4^Psychological Neuroscience Lab, CIPsi, School of Psychology, University of Minho, Braga, Spain; ^5^Departamento de Psicología Básica, Clínica y Psicobiología, Universitat Jaume I, Castelló de la Plana, Spain

**Keywords:** COVID-19, psychological impact, obsessive-compulsive symptoms, general population, adaptation

## Abstract

**Introduction:**

Stressful events are one cause for the emergence and/or worsening of obsessive-compulsive symptoms. The public health measures employed to prevent the contraction of the COVID-19 virus overlap with common behaviors adopted by people diagnosed with obsessive-compulsive disorder. Thus, we decided to study the longitudinal impact of the pandemic in the general Portuguese population assessed with the Obsessive-Compulsive Inventory (OCI-R), and the Depressive, Anxiety, and Stress Scale (DASS-21).

**Methods:**

One hundred and eighty-nine participants reported their scores at three different time-points of the pandemic in Portugal: March of 2020, March of 2021, and March of 2022. Non-parametric repeated measures analyses were performed on the OCI-R and DASS-21 scores to analyze differences in the levels of symptomatology throughout time.

**Results:**

We found statistically significant differences with time in the OCI-R total and washing subscale scores, as well as in the anxiety subscale of DASS-21 score. For OCI-R total, we found significantly higher scores in 2020 compared to 2021 and 2022, and for the washing subscale we found statistically significant decreases with time. In terms of anxiety scores, we found significantly lower symptoms in 2021 compared to the others.

**Conclusion:**

The reliance on the washing-like behaviors to contain the pandemic spreading explains its augmented scores in the acute phases of the pandemic and thus the continuous decrease of symptomatology with time. For anxiety, both the beginning and the end of the pandemic seem to have posed a threat, leading to an increase in worry and hypervigilance. In general, our results demonstrate the adaptative nature of humans and the instrumental role of psychological distress to cope with the world around us.

## 1 Introduction

The COVID-19 pandemic led to a worldwide state of crisis with a significant impact on the mental health of the general population. The literature has been documenting extensively the surfacing of acute mental health responses to this pandemic, expressed mainly through symptoms of anxiety, depression, and stress (Silva Moreira et al., 2021; Picó-Pérez et al., 2021; Costa et al., 2022). However, stressful events are also one of the possible causes for the onset and/or worsening of obsessive-compulsive (OC) symptoms (Fontenelle et al., 2021). Coupled with the widespread panic associated with the fear of contracting this new virus, the absence of knowledge about its transmission, the lack of treatment options during the first waves of this pandemic, and the further reliance on public health measures such as washing hands and refraining from touching possible contaminated objects to contain the spreading of the virus, there were particularly vulnerable times to the expression of obsessive-compulsive symptoms in clinical (Guzick et al., 2021) and non-clinical samples (Fontenelle et al., 2021). Indeed, the literature reveals that the burden posed by this pandemic on people previously diagnosed with mental health disorders affected particularly those people suffering from Obsessive-Compulsive Disorder (OCD). One potential explanation for this is the overlap between the public measures implemented and OCD symptoms, making it—challenging to determine if someone presenting the behaviors (i.e., compulsions), was also developing a maladaptive interpretation of their thoughts and situations (i.e., obsessions) (Fontenelle and Miguel, 2020; Linde et al., 2022; Banerjee, 2020).

The intolerance to uncertainty, a trait commonly expressed by people with OCD, may contribute to this additional difficulty when facing such unprecedented times. Moreover, the stakeholders’ messages, constantly covered in media reports, conveying this need for hypervigilance and assurance-seeking behaviors regarding hygiene and precautionary health measures, may explain why within all dimensions of OCD, contamination and checking related symptoms have been demonstrating special relevance and association with poorer outcomes during the pandemic context (Linde et al., 2022). To deal with the insecurity and the illness unfamiliarity, people may compensate by extreme adherence to public health measures and complete isolation, contributing to the burden on their mental health (Tolin et al., 2003). General public’s inexperience with these behaviors may also explain the additional hurdle of adhering to and accepting the changes caused by this novel threat, representing heightened stress responses when compared with people diagnosed with OCD (Perkes et al., 2020).

To some degree, we can understand the adaptative role of the employment of this type of behavior to fight a pandemic without precedence (Fontenelle and Miguel, 2020). It seems that greater COVID-related fear and concerns are associated with more prevalence of these obsessive-compulsive symptoms (Tolin et al., 2003). Furthermore, the employment of these contamination preventive measures is also referred to as the cause for more worry and anxiety associated with the pandemic (Ojalehto et al., 2021), making it difficult to disentangle if the OC-related behaviors are expressed as the result of the insecure context installed worldwide or if they created a mechanism of positive feedback in which both COVID-19 related fear and OC symptoms are mutually exacerbating each other.

Given that all people have been implementing in their routines behaviors typically employed by people with OCD in response to this new stressor, we would like to understand whether the COVID-19 pandemic had such a deleterious impact on people’s mental health status, leading to the emergence of psychological symptoms in the general population, and consequently, to lasting changes in the way their world is perceived. Therefore, our study aims to analyze the presence of psychological distress and OCD-related symptoms in the general population, as well as their trajectory during 3 years of living with COVID-19 restrictions. We expect to find higher levels of OCD-related symptoms in the first waves/acute phases of the pandemic as compared to later times, reflecting the instrumental role that those types of symptoms may have represented in the safety of people. Similarly, the same pattern is hypothesized to occur for depression, anxiety, and stress, considering an acute response to the novelty of the stressor, followed by an adaptation through time.

## 2 Materials and methods

From an initial Portuguese sample of 2040 adult subjects (detailed methods described in Silva Moreira et al., 2021 and Picó-Pérez et al., 2021), 219 participants responded to the Obsessive-Compulsive Inventory-Revised (OCI-R; Cunha et al., 2022; Foa et al., 2002) and the Depression, Anxiety and Stress Scale (DASS-21; Pais-Ribeiro et al., 2004; Lovibond and Lovibond, 1995) at three time points (2020, 2021, and 2022).

The OCI-R is a self-report scale consisting of 18 items that assess the severity of the OC symptoms in the previous month on a 5-point Likert scale ranging from “Not at all” (0) to “Extremely” (Fontenelle et al., 2021). It has a Total score (Cronbach’s α* = 0.888) and six subscales that represent different symptom dimensions, namely Washing (Cronbach’s α* = 0.770), Checking (Cronbach’s α* = 0.713), Obsessing (Cronbach’s α* = 0.798), Neutralizing (Cronbach’s α* = 0.692), Ordering (Cronbach’s α* = 0.817), and Hoarding (Cronbach’s α* = 0.747).

DASS-21 is a psychometric scale comprising 21 items designed for assessing three types of psychological symptoms: depression, anxiety, and stress. It also provides a total score, with higher scores indicating more severe symptomatology experienced in the preceding week.

In this study, the OCI-R and DASS-21 were applied through an online survey using Google Forms and were used as repeated measures to assess the psychological impact of COVID-19. The initial data collection occurred in March 2020, shortly after the beginning of the first mandatory confinement in Portugal. For the purposes of this study, one-year intervals were considered for repeated measures since the onset of the pandemic in Portugal (i.e., March 2020, March 2021, and March 2022). In March of 2021, about 2 months had elapsed after the implementation of a new period of confinement in our country and in March of 2022, about one-month had passed since the public health measures in Portugal began to gradually ease.

Due to the non-normality of the data, Friedman tests, non-parametric analysis of repeated measures, were performed on the OCI-R and DASS-21 scores to analyze differences in the levels of symptomatology throughout time. These were followed by post hoc analyses to understand the specific time points driving the statistical differences. All statistical analyses were performed with the JASP software; (Version 0.18.3; JASP Team, 2024). *p* values under 0.05 were considered statistically significant.

All the study procedures here described followed the ethical requirements for human research in agreement with the Declaration of Helsinki and were accordingly approved by the Ethical Committee for Life Sciences of the University of Minho.

## 3 Results

Two hundred and nineteen participants responded to our online forms reporting their psychological symptoms measured by OCI-R and DASS-21 in all three timepoints, namely March 2020, March 2021, and March 2022. However, 30 of them reported the existence of a psychiatric diagnosis and thus were excluded from the analysis. A final sample of 189 was considered for the purpose of this study. The subsample (*N* = 189, 85.81% female) had a mean age of 39.06 (SD = 11.53) years old and 18.16 (SD = 3.27) mean education years.

**Figure 1:**
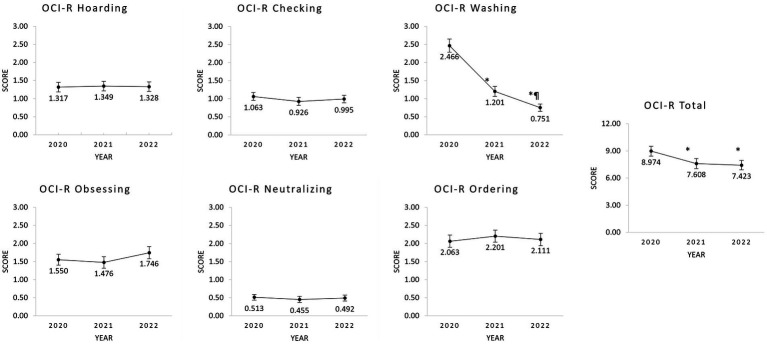
Plots representing the mean values and standard error of the OCI-R subscales on the three timepoints studied. *Significant difference in the Conover’s post-hoc test in comparison to 2020. ¶Significant difference in the Conover’s post-hoc test in comparison to 2021. OCI-R, Obsessive-Compulsive Inventory—Revised.

Regarding the OC symptoms across time, upon examining Figure 1, we can observe a maintenance of the symptomatology levels for all subtypes of symptoms, except for washing and total scores in which a decrease can be seen. In fact, when applying repeated-measures analyses on all the OCI-R subscales, there were statistically significant differences for the OCI-R total [*χ*^2^(3) = 16.035, *p* < 0.001, Kendall’s W = 0.042] and washing subscale [*χ*^2^(3) = 137.953, *p* < 0.001, Kendall’s W = 0.365], but not for the other five subscales of the OCI-R. Regarding the post-hoc tests, in the OCI-R total, there were statistically significant differences between 2020 and 2021 (Z = 3.595, pholm = 0.001), and 2020 and 2022 (Z = 3.327, pholm = 0.002), while for the washing symptoms, there were statistically significant differences between all time points: 2020 vs. 2021 (Z = 8.414, pholm < 0.001), 2020 vs. 2022 (Z = 11.328, pholm < 0.001), and 2021 vs. 2022 (Z = 2.914, pholm = 0.004). In both OCI-R total and washing subscores, the highest score was registered in 2020.

**Figure 2:**
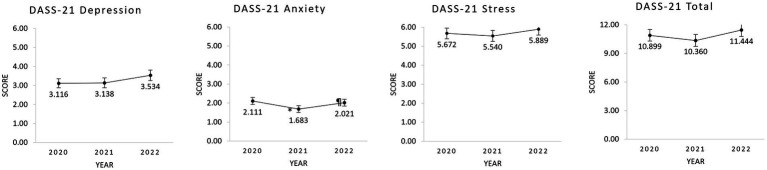
Plots representing the mean values and standard error of the DASS-21 subscales on the three timepoints studied. *Significant difference in the Conover’s post-hoc test in comparison to 2020. ¶Significant difference in the Conover’s post-hoc test in comparison to 2021. DASS-21, Depression, Anxiety, and Stress Scale-21.

Considering the psychological symptoms assessed with the DASS-21 scale, looking at Figure 2, we can observe approximately the same levels of symptomatology for all the subscales, with only the depression scores deviating from a V pattern. When assessing changes of these symptomatology with time through repeated-measures analyses, there were only statistically significant differences in the anxiety subscale [*χ*^2^(3) = 7.258, *p* = 0.027, Kendall’s W = 0.019]. Post-hoc analysis revealed that anxiety symptoms in 2021 were significantly lower than in 2020 (Z = 2.561 pholm = 0.011) and in 2022 (Z = 2.012, pholm = 0.045).

## 4 Discussion

At a descriptive level, upon examining Figure 1, we can observe higher scores of washing and total OC symptoms in the acute phase of the pandemic in Portugal (i.e., March 2020), that continuously decreased with time. When observing the other subscales of OCI-R on Figure 1, approximately the same levels of symptomatology can be seen throughout the pandemic in Portugal, which is contrary to what we hypothesized In Figure 2, when observing all the subscales of DASS-21, approximately the same V pattern of symptoms is seen in all subscores, except for depression, where we do not see the deflection in 2021 compared to the other two timepoints.

In this line, when performing a repetitive-measures analysis of the symptomatology assessed with OCI-R and DASS-21, we found statistically significant differences with time on the washing subscale and total score of OCI-R, and also in the anxiety subscale of DASS-21. For both OCI-R subscales, we found significantly higher scores in 2020 compared to the other two time-points and for the washing subscale, the score obtained in 2021 was also significantly higher than in 2022. On the other hand, for the anxiety subscale of DASS-21, the scores obtained in 2021 were significantly lower than in 2020 and in 2022.

Given the similar pattern of findings for OCI-R total and washing symptoms, we can assume that the statistically significant changes found in the total scale are driven by the changes found in the washing subscale. In fact, due to the nature of the public health measures enforced during the acute phases, it was expected that this type of symptoms would be more prevalent than others (Linde et al., 2022). Considering the widespread panic and safety-seeking behaviors observed globally, it was suspected that initial levels of washing-like symptomatology would be significantly inflated even in non-clinical samples (Fontenelle et al., 2021), explaining our significant decrease over time. The continuous decrease observed in the washing scores indicates that the exhaustive employment of contamination preventive-like behaviors that could lead to the emergence of a very chronic form of psychopathology was not further maintained. Interestingly, other reports from the first year of the pandemic seemed to indicate that OC symptoms did not decrease (Loosen et al., 2021), which did not prove to be the case when analyzing cohorts during a longer period, as presented in this study. Moreover, the analysis of the pattern of variation in washing obsessive symptoms seems to highlight an adaptive response across time and not necessarily a pathological manifestation of the disease (Polimeni et al., 2005). As proposed in Meșterelu (Meșterelu et al., 2021), OC symptoms may have served as a protective element of health of these sample on the beginning of the pandemic, ensuring the adherence to protective measures, with a subsequent decrease in later stages of the pandemic when these behaviors were not that necessary.

Furthermore, for the anxiety subscale of DASS-21, the V pattern seen highlights a significant decrease of the levels of anxiety from the beginning of the pandemic (i.e., 2020) to 2021, followed by an increase in 2022. These results show a normative response of our Portuguese sample to threats (Fernandes et al., 2024). In the acute phase (2020) and during the lifting of the COVID restrictions in Portugal (2022), people seem to have evidenced augmented worry and agitation as assessed by the anxiety subscale of DASS-21, while in 2021 a habituation response seems to be at play, considering nothing had changed in the contextual pandemic setting compared to 2020. Therefore, as in 2022 the symptoms increase again, the absence of knowledge about the pandemic is hypothesized to pose the same burden as the return to life previous to COVID. This may be explained by hypervigilance to possible negative health outcomes, giving the stopping of the use of face mask, among other restrictions. However, other complex and devastating crisis events like the beginning of the war in Ukraine (Massag et al., 2023) may be accounting for the increased distress suffered by the Portuguese population around the time of the lifting of COVID restrictions in 2022.

The longitudinal design of our study made it possible to describe the continuous effect of the COVID-19 pandemic on the Portuguese population mental health. The pattern observed in this sample is consistent with the adaptation to a new reality, in which people were overall confined to their houses and informed to rely on repetitive and restrictive hygiene rituals to ensure their safety (Tolin et al., 2003). The mental health status’ changes observed in the general public throughout these 3 years living within a pandemic framework seem to have been maintained only due to their instrumental role. The obsessive-like coping mechanisms reinforced by the governments and public health specialists did not appear to have become problematic after the removal of the threat. Contrary to what was found with previous epidemics, there did not seem to appear a delayed development of symptoms of poor mental health (Banerjee, 2020). It seems that although people engaged in OC-like behaviors, the cognitive bias that typically accompanies the compulsions in OCD may not have been developed, being the actions seen as a real need to a very real threat, rather than an overestimation of control over events or a biased interpretation of mainstream situations as dangerous (Grant et al., 2022). This interpretation remains speculative though, since we did not directly measure potential cognitive biases associated with OC symptomatology. Additionally, the increased focus on cleanliness during the pandemic might have provided a sense of validation or acceptance for individuals with contamination-like symptoms. This normalization could have potentially led to a decrease in distress associated with such symptoms.

In general, the results of this study yield some positive outcomes related to the pandemic in Portugal. In a 3-year longitudinal framework, there were no observed nefarious and pervasive alterations in the mental health of the general Portuguese population. These findings should be generalized to other populations and cultural contexts with cautious and in light of the public health measures applied, the level of knowledge conveyed by governments, and the timing of the access to vaccination in each country, in order to characterize properly the responses of people to this continuous stressor. Although our work captures a general longitudinal trajectory of symptoms displayed by our sample, it does not reflect the heterogeneity of the mental health implications of facing a pandemic. It is possible that some people may have increased vulnerability to developing mental health disorders in response to stressors, thus, possibly still expressing an intense response to COVID-19 in 2022. Future studies could employ a data-driven approach (Fernandes et al., 2024), to identify vulnerability profiles and portray a more comprehensive description of the psychological impact of the COVID-19 pandemic. Despite the growing body of literature pertaining to the pandemic, many lessons and topics were brought to the attention of the world governates, demonstrating the relevance of continuing to publish articles on the subject. The globalized experience of undergoing such stressful events resulted in a greater valorization of one’s mental health, highlighting the need to consider the psychosocial dimension of health, which had been disregarded in public health policies and speeches.

## Data Availability

The original contributions presented in the study are included in the article/supplementary material, further inquiries can be directed to the corresponding authors.
